# Sweet cherry fruit cracking: follow-up testing methods and cultivar-metabolic screening

**DOI:** 10.1186/s13007-020-00593-6

**Published:** 2020-04-10

**Authors:** Michail Michailidis, Evangelos Karagiannis, Georgia Tanou, Eirini Sarrou, Katerina Karamanoli, Athina Lazaridou, Stefan Martens, Athanassios Molassiotis

**Affiliations:** 1 Laboratory of Pomology, School of Agriculture, Aristotle University of Thessaloniki, 570 01, Thessaloniki-Thermi, Greece; 2 Institute of Soil and Water Resources, ELGO-DEMETER, Thessaloniki, 57001 Greece; 3 Institute of Plant Breeding and Genetic Resources, ELGO-DEMETER, Thessaloniki, 57001 Greece; 4 Laboratory of Agricultural Chemistry, School of Agriculture, Aristotle University of Thessaloniki, Thessaloniki, Greece; 5 Laboratory of Food Chemistry – Biochemistry, Dept. of Food Science & Technology, Faculty of Agriculture Aristotle University, Thessaloniki, Greece; 6 Department of Food Quality and Nutrition, Centro Ricerca e Innovazione, Fondazione Edmund Mach, 38010 San Michele all’Adige, Trento, Italy

**Keywords:** Cracking, Fruit, Primary metabolites, Secondary metabolites, Skin tissue, Sweet cherry cultivars

## Abstract

**Background:**

Rain-induced fruit cracking is a major physiological problem in most sweet cherry cultivars. For an in vivo cracking assay, the ‘Christensen method’ (cracking evaluation following fruit immersion in water) is commonly used; however, this test does not adequately simulate environmental conditions. Herein, we have designed and evaluated a cracking protocol, named ‘Waterfall method’, in which fruits are continuously wetted under controlled conditions.

**Results:**

The application of this method alone, or in combination with ‘Christensen method, was shown to be a reliable approach to characterize sweet cherry cracking behavior. Seventeen cherry cultivars were tested for their cracking behavior using both protocols, and primary as well as secondary metabolites identification was performed in skin tissue using a combined GC–MS and UPLC-MS/MS platform. Significant variations of some of the detected metabolites were discovered and important cracking index–metabolite correlations were identified.

**Conclusions:**

We have established an alternative/complementary method of cherry cracking characterization alongside to Christiansen assay.

## Background

Sweet cherry (*Prunus avium* L.) is an important temperate fruit crop and its production is characterized by short duration of fruit development during spring until the middle of summer in North hemisphere. Climate change predictions (IPCC, 2013) point to an increasing frequency of excessive rainfall in the early-middle summer to the Eurasian zone [1] that will result to an increasing of the sweet cherries fruit cracking. Particularly, in sweet cherry, rain-induced cracking before harvest is the most significant crop loss in many cherry-producing areas with enormous commercial losses worldwide [2]. This physiological disorder is developed as cracks of the fruit skin after rainfall, sometimes deep into the flesh, affecting the stem end area, the calyx end and the cheeks of the fruit (side cracks) [3]. Although sweet cherry cracking has been investigated for many years, few advances have been made in understanding the metabolic basis of fruit cracking susceptibility in the various cultivars [2].

The basic mechanism that causes skin cracking in sweet cherries, although not completely elucidated, focuses on the rapid increase of water absorption by the fruit either by direct absorption from the skin of the fruit or by the absorption of water through the tree vascular system [4]. In general, three types of cherry splitting have been described in the literature: stem end cracks, top end cracks and common lateral cracks [5]. The type of split possible is determined by the occurrence of different way of water uptake [6].

The cherry fruit peel includes a thin layer of epidermis (1 μm) and up to eight layers of cells, with an overall thickness of 4.5 μm [7]. The epidermis is consisted of external hydrophobic substances and an inner layer of hydrophilic substances (polyurines and glucans), with a single epidermal layer of cellulose. The density of fruit stomata (85–200 per cm^2^), is lower in comparison to the leaves (5000–10,000 per cm^2^) [8], however, fruit contain enough pores that are permeable to water [9].

In order to classify sweet cherry cultivars into relative sensitive, moderate and resistant to cracking, the cracking index was traditionally used [5] with minor modifications. The cracking index was determined by immersing the fruits in distilled water for a certain duration and then fruits were observed for surface splits [5], briefly from now it will be referred as ‘Christensen method’. In this in vivo cracking assay, however, the immersion of the fruits in water does not reflect the actual field conditions, where the entire fruit skin surface does not receive the same water pressure, as it occurs during the application of Christensen method [6]. For this purpose, in this work, an alternative method for determining the cherry fruit cracking index has been developed. In this assay, herein referred as ‘Waterfall method’, the fruits are not immersed in distilled water, but covering fruits are continuously wetted with deionized water; such conditions are more accurately simulate the water pressure on the fruit surface as well as the water absorption by fruit skin during rain.

Although information regarding skin tissue metabolism in sweet cherries is scarce, recent studies investigated the sequence of events accounts for rain cracking in sweet cherry [10, 11]. Based on the above-mentioned studies, it is well accepted that an early metabolic reprogramming occurs in cherry fruit prior to cracking events; however, yet no clear consensus exists on which compounds act as possible cracking biomarkers. Accordingly, the aim of this study was to compare the two methods, namely Christensen and Waterfall method, for determining the fruit cracking index using seventeen sweet cherry cultivars that displayed different cracking behavior. An additional purpose of this work was to explore the metabolic changes in these cultivars and the possible association with their cracking features obtained by two assays. Thus, this work has the potential to provide a useful assay method for fruit cracking evaluation and a large-scale sweet cherry skin metabolome monitoring analysis under cracking process.

## Materials and methods

### Plant material and sampling procedure

**Fig. 1:**
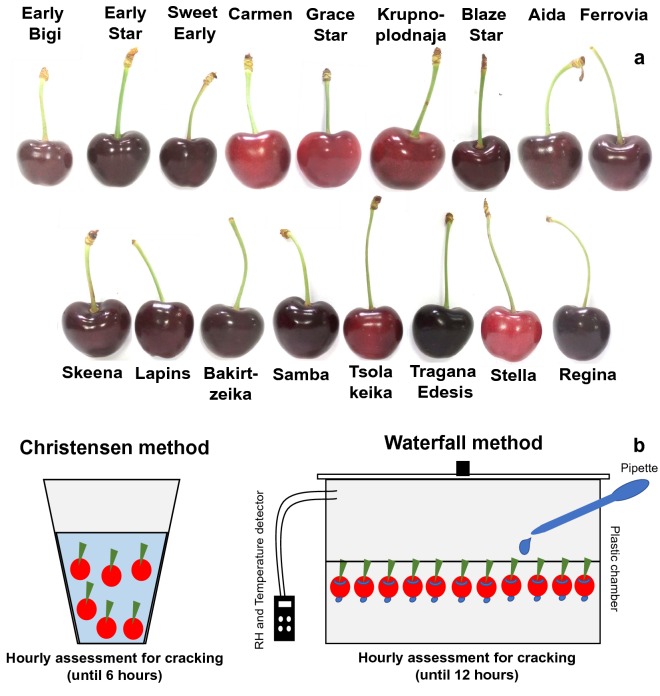
Plant material and experimental design used. **a** Phenotypes of seventeen sweet cherry cultivars harvested at commercial ripening stage. **b** Graphical presentation of two applied methods (Christensen and Waterfall) for the determination of skin cracking index in sweet cherry fruits

Fruits of seventeen sweet cherry cultivars at commercial harvest stage were collected (Fig. 1a) and evaluated for susceptibility to fruit cracking. The experiment was conducted in an experimental sweet cherry orchard of the Farm of Aristotle University of Thessaloniki (Thermi, Thessaloniki) and in a commercial orchard (Agras, Pellas region) during 2017 growing season. The tested cultivars were ‘Early Bigi’, ‘Early Star’, ‘Sweet Early’, ‘Carmen’, ‘Grace Star’, ‘Krupnoplodnaja’, ‘Blaze Star’, ‘Aida’, ‘Ferrovia’, ‘Skeena’, ‘Lapins’, ‘Bakirtzeika’, ‘Samba’, ‘Tsolakeika’, ‘Tragana Edesis’, ‘Stella’ and ‘Regina’. The two orchards consisted of 14-years old trees, planted at 5 × 5 m spacing between rows and along the row, grafted onto ‘Mahaleb’ rootstock, trained in open vase and subjected to standard cultural practices. Fruit were picked at full maturity based upon size, color, and commercial picking dates in each area. About one thousand fruits of each cultivar were harvested, 60 fruits of them randomly were divided into three 20-fruit sub-lots, then the skin was separated from flesh and thereafter the skin was immersed in liquid nitrogen and stored at − 80 °C for metabolites analysis. Experiment was following completely randomized design (CRD). Particularly, we used 5 replicates of 15 fruits for each cracking method; 150 fruits for the fruit water absorption and the main cracking observations; 5 replicates of 10 fruits for the physio-biochemical traits; 30 fruits for the textural properties of the skin.

### Cracking estimation methods

Susceptibility of the sweet cherries to fruit cracking was estimated using the Christensen and Waterfall methods, in order to evaluate the fruit cracking index. A graphical representation of both methods is shown in Fig. 1b, while a description of these assays is outlined below.

Christensen method: Skin cracking was determined as described with some modifications [5]. Fruits of each cultivar (5 replicates × 15 fruits) were weighted and immersed in distilled water under room temperature conditions for a total period of 6 h with parallel hourly recording for cracking incidence. The results were expressed as percentage of cultivar cracking susceptibility by the following equation:

$$\left[ {\mathop \sum \limits_{i = 1}^{6} \left( {7 - {\text{i}}) \times {\text{cracked fruits in i hour}}} \right) / 6\, \times \,{\text{total fruits}}} \right] \, \times { 1}00.$$

According to the fruit cracking index values obtained with Christensen method, all cultivars were divided into four groups: low susceptible (cracking index lower than 10.0), moderately susceptible (cracking index from 10.1 to 30.0), susceptible (cracking index 30.1 to 50.0) and highly susceptible (cracking index > 50.1) [5].

Waterfall method: Fruits of each cultivar (5 replicates ×  15 fruits) were weighted and placed to hang from the stem in a transparent plastic chamber (56 × 39 × 28 cm) with a temperature/relative humidity data logger (HOBO H08-003-02). Drops of distilled water were pipetted (3 mL plastic pipette) on the hovering sweet cherry fruits for a total period of 12 h with parallel hourly recording for cracking incidence the first 4 h and 2-h recording thereafter. The results were expressed as percentage of cultivar cracking susceptibility by the modified equation:

$$\left\{ {\left[ {\mathop \sum \limits_{i = 1}^{4} \left( {13 - {\text{i}}) \times {\text{cracked fruits in i hour}}} \right) + \mathop \sum \limits_{n = 3}^{6} \left( {13 - 2 \times {\text{n}}) \times {\text{cracked fruits in }}\left( {2 \times {\text{n}}} \right) {\text{hour}}} \right)} \right] \, /{ 12 } \times {\text{ total fruits}}} \right\} \, \times { 1}00.$$

### Physio-biochemical traits of cherry fruit

Determination of physiological traits of each cultivar, namely fresh weight of ten fruits and percentage fresh weight of stem, flesh and skin in five replicates of ten fruits were performed at harvest (Additional file 1: Table S1). Fifty fruits were randomly divided into five 10-fruit sub-lots, and analyzed for total soluble solids (TSS), titratable acidity (TA) and external color (Chroma and Hue angle) at harvest as described [12].

### Textural properties of skin tissue

Skin penetration (in N) for each cultivar (thirty fruits) was performed at harvest using a TA-XT2i Texture Analyzer (Stable Microsystems, Godalming, Surrey, UK) as described in detail [13] following a four sides skin penetration protocol (upper side, suture, apical and near stem) (Additional file 2: Table S2). Total skin penetration was calculated as the average of four sides measurements (Additional file 2: Table S2).

### Fruit water absorption

Sweet cherry fruits were weighted before coming in touch with water and then just after the end of each method (Christensen and Waterfall methods) after removal of surface water by centrifugation and air flow for 1 min. The results were expressed as % percentage of fruit water absorption.

### Observations for main cracking

Just after the end of Christensen and Waterfall methods for cracking estimation, the four sides of fruits (upper side, suture, apical and near stem) were observed to depict the main type of cracking for each cultivar (Additional file 2: Table S2). In each cultivar, side or sides of fruits with a significant difference among sides based on Duncan’s Multiple Range Test, they represent the main type of skin cracking.

### Primary metabolites analysis in skin tissue by GC–MS

Frozen grinding skin tissue (0.3 gr) of cultivars at harvest, was transferred in 2-mL screw cap tubes. Determination of primary polar metabolites was conducted as described with slight modifications [14]. In sample, 1400 μL methanol and 100 μL adonitol (0.2 mg mL^−1^) was added and incubated for 10 min at 70 °C. The supernatant after centrifugation was reserved and 750 μL chloroform and 1500 μL dH_2_O were added. 150 μL of the upper polar phase was dried, re-dissolved in 40 μL methoxyamine hydrochloride (20 mg mL^−1^, 120 min, 37 °C), derivatized with 70 μL *N*-methyl-*N*-(trimethylsilyl) tri-fluoroacetamide reagent (MSTFA) and incubated (30 min, at 37 °C). The GC–MS analysis was carried with a Thermo Trace Ultra GC equipped with ISQ MS and TriPlus RSH autosampler (Switzerland) [15]. Samples (1 μL) was injected and split ratio was 70:1. A TR-5MS capillary column 30 m × 0.25 mm x 0.25 μm was used. Injector temperature was 220 °C ion source 230 °C and the interface 250 °C. Carrier gas flow rate was 1 mL min^−1^. Temperature program was held at 70 °C for 2 min, then increased to 260 °C (rate 8 °C min^−1^), where it remained for 18 min, m/z50–600 was recorded. Standards were used for peak identification or NIST11 and GOLM metabolome database (GMD) in case of unknown peaks [16]. The detected metabolites were assessed based on the relative response compared to adonitol and expressed as relative abundance (Additional file 3: Table S3). Experiments performed using three biological replicates.

### Secondary metabolites analysis in skin tissue by UPLC–MS/MS

Sweet cherries skin polyphenolic extraction in cultivar fruits at harvest was performed as previously described [17]. Freeze dried sweet cherry (100 mg) tissue (mesocarp-exocarp) was mixed with 4 mL methanol (80%) into a 15-mL falcon tube. The mixture was sonicated (20 min), shaken (3 h, 20 °C) and incubated at 4 °C (overnight) in the dark. Secondary metabolites extract was acquired following filtration through a 0.22 µm PFTE membrane into a glass vial.

Targeted ultra-performance liquid chromatography-tandem mass spectrometer (UPLC–MS/MS) was performed on a Waters Acquity system (Milford, MA, USA) consisting of a binary pump, an online vacuum degasser, an autosampler, and a column compartment. Separation of the phenolic compounds was achieved on a Waters Acquity HSS T3 column 1.8 μm, 100 mm × 2.1 mm, kept at 40 °C. The phenolic analysis was performed, as previously described [18]. Anthocyanins were quantified using the method previously described [19, 20] on a RP Acquity UPLC^®^ BEH C18 column (130 Å, 2.1 × 150 mm, 1.7 µm, waters), protected with an Acquity UPLC^®^ BEH C18 pre-column (130 Å, 2.1 × 5 mm, 1.7 µm, Waters).

Mass spectrometry detection was carried out on a Waters Xevo TQMS instrument equipped with an electrospray (ESI) source. Data processing was carried out using the Mass Lynx Target Lynx Application Manager (Waters). Three biological replications were used for each cultivar. The datasets used and/or analyzed during the current study are available from the corresponding author on reasonable request.

### Statistical analysis

Statistical analysis of 17 cultivars was conducted using SPSS (SPSS v23.0., Chicago, USA) by multivariate analysis of variance (MANOVA) and provided in Additional Tables. Mean values of all cultivars tested were compared by the least significance difference (LSD) for quality traits based on 5 biological replicates of 15 fruits, apart from textural properties for which 30 individual fruits were used (P ≤ 0.05). For metabolite analysis, 3 independent biological replicates (P ≤ 0.05) were performed. The cultivars were grouped based on the two cracking estimation methods using hierarchical cluster analysis (nearest neighbor, squared euclidian distance). Spearman correlation analysis of all cultivars among cracking indexes and other variables was also performed (Additional file 4: Fig. S1).

## Results

### Cultivars fruit quality traits at harvest

**Fig. 2:**
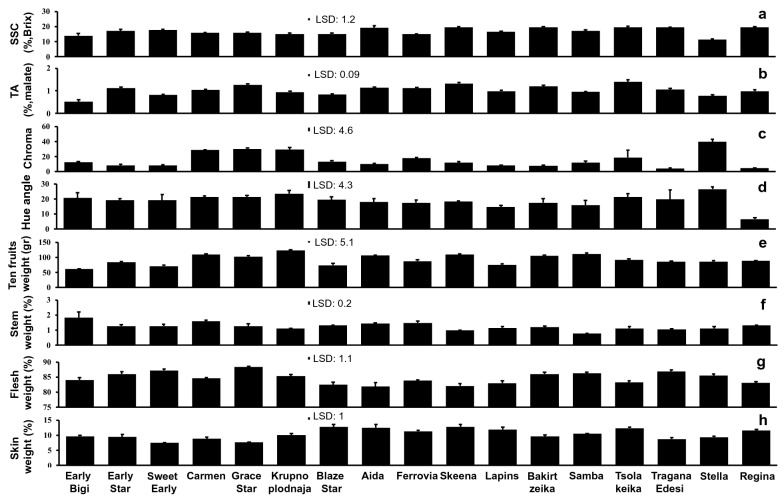
Physiological characterization of the various sweet cherry cultivars. Determination of soluble solids concentration (SSC) **a**, titratable acidity (TA) **b**, color index Chroma **c**, color index Hue angle **d**, weight of ten fruits **e**, weight of stem (%) **f**, weight of flesh (%) **g**, weight of skin (%) **h**. Each value represents the mean of five replications x ten fruits. Vertical bars represent SD. Differences among cultivars detected based on least significant difference (LSD); P ≤ 0.05. Arithmetic data are provided in Additional file 1: Table S1

To study the sweet cherry fruit cracking responses, we selected seventeen representative and frequently planted cultivars in Greece. Soluble solids concentration (SSC) of sweet cherry fruits at ripening stage (Fig. 1a) ranged from 11.27 (‘Stella’) to 19.63 (‘Skeena’) Brix’s percentage (Fig. 2a). The acidity of cultivars ranged from 0.51 to 1.4 (%, malate), the lowest and the highest acid concentration was detected for ‘Early Bigi’ and in ‘Tsolakeika’, respectively (Fig. 2b). The color indexes, Chroma and Hue angle of cherries ranged from 3.9 (‘Tragana Edesis’) and 6.6 (‘Regina’) to 39.5 (‘Stella’) and 27.0 (‘Stella’), respectively (Fig. 2c, d). The average weight (g) of ten fruits per cultivar ranged from 60.9 to 123.9, the minimum weight was recorded for ‘Early Bigi’ and the maximum for ‘Krupnoplodnaja’ (Fig. 2e). The relative weight of stem, flesh and skin, expressed as partitioned  % percentage of whole cherry fruits, were ranged in stem (Fig. 2f) from 0.8 (‘Samba’) to 1.8 (‘Early Bigi’), in flesh (Fig. 2g) from 82.1 (‘Skeena’) to 88.3 (‘Grace Star’) and in skin (Fig. 2h) from 7.5 (‘Sweet Early’) to 12.8 (‘Skeena’).

### Texture properties of the skin tissue

**Fig. 3:**
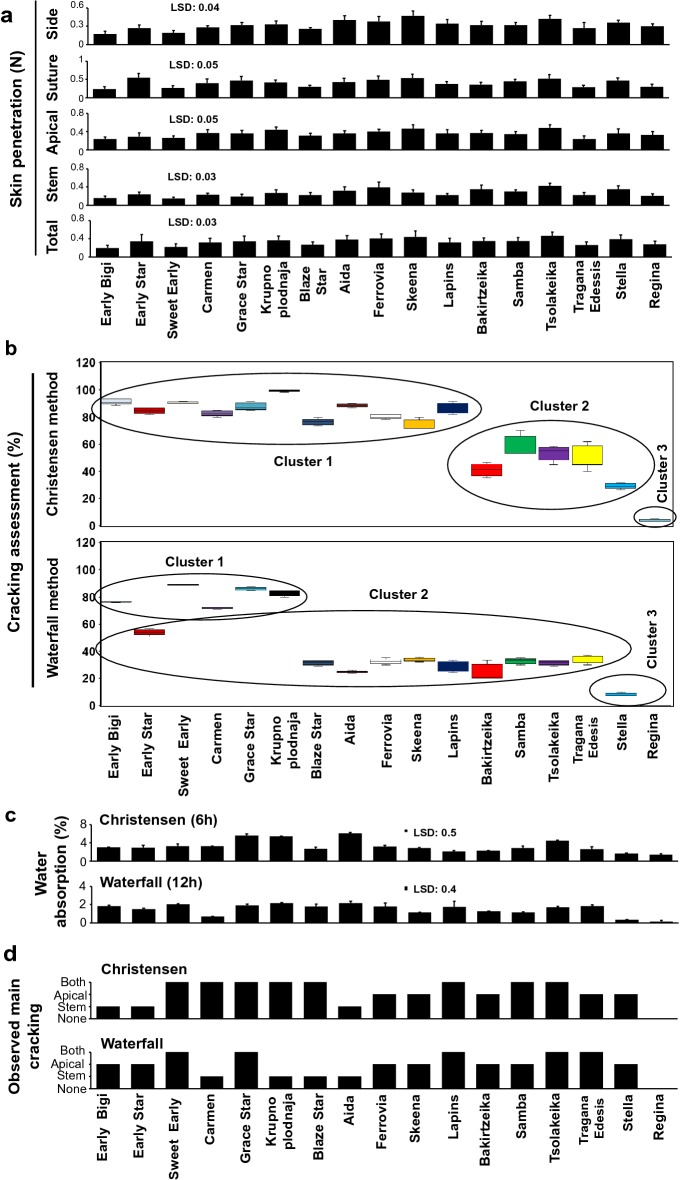
Cracking-related features of sweet cherry cultivars in response to Christensen and Waterfall method. Texture properties of the various sweet cherry cultivars expressed as skin penetration force at side, suture, apical, stem and total **a**. Fruit cracking index tested with Christensen and Waterfall method **b**. Fruit water absorption (%) as evaluated by Christensen and Waterfall methods **c**. The observed main cracking classes (none = zero, stem = one, apical = two, and both stem and apical = three) of sweet cherry cultivars subjected to Christensen and Waterfall methods **d**. Each value represents the mean of 5 replications × 15 fruits. Vertical bars represent SD. Means of cultivars are compared with least significant difference (LSD; P ≤ 0.05). Boxplot and cluster analysis of 17 cultivars was performed on fruit cracking index with Christensen and Waterfall method. Arithmetic data are provided in Additional file 2: Table S2

Side skin penetration force among cultivars was ranged from 0.17 (‘Early Bigi’) to 0.47 N (‘Skeena’) (Fig. 3a). Suture skin penetration force was ranged from 0.24 (‘Early Bigi’) to 0.55 N (‘Early Star’). Also, the apical skin penetration force was ranged from 0.24 (‘Early Bigi’ and ‘Tragana Edesis’) to 0.48 N (‘Tsolakeika’). Stem skin penetration force were ranged from 0.15 (‘Sweet Early’) to 0.42 N (‘Tsolakeika’). Finally, the total skin penetration force was ranged from 0.2 (‘Early Bigi’) to 0.46 N (‘Tsolakeika’) (Fig. 3a).

### Cracking index and fruit water absorption of cherry cultivars

The application of both cracking methods (Christensen and Waterfall) showed that the ‘Regina’ was the most resistant to skin cracking among the cultivars tested (Fig. 3b). Based on Christensen method, eleven cherry cultivars were evaluated as susceptible to cracking, with cracking index higher than 70% (Fig. 3b). These relative cracking-sensitive cultivars include ‘Early Bigi’, ‘Early Star’, ‘Sweet Early’, ‘Carmen’, ‘Grace Star’, ‘Krupnoplodnaja’, ‘Blaze Star’, ‘Aida’, ‘Ferrovia’, ‘Skeena’ and ‘Lapins’. Using the Waterfall method, five cultivars namely ‘Early Bigi’, ‘Sweet Early’, ‘Carmen’, ‘Grace Star’ and ‘Krupnoplodnaja’ with cracking index higher than 50% were identified (Fig. 3b) based on cluster analysis. Furthermore, sharp absorption of water in ‘Grace Star’, ‘Krupnoplodnaja’, ‘Aida’ and ‘Tsolakeika’ where exceeding 4% of the total fruits weight based on the Christensen method (Fig. 3c). Three cultivars (‘Sweet Early’, ‘Krupnoplodnaja’ and ‘Aida’) with water absorption higher than 2% of total fruit weight was found using the Waterfall method (Fig. 3c). Differences between the two tested methods concerning the type of cracking were also documented (Fig. 3d). For instance, to the cultivars ‘Early Bigi’ and ‘Early Star’ the main type of cracking was near the stem based on Christensen method (Fig. 3d); on the contrary, in the same cultivars the main type of cracking was the apical according to Waterfall method (Fig. 3d).

### Cultivar-specific primary metabolic profile of skin tissue

**Fig. 4:**
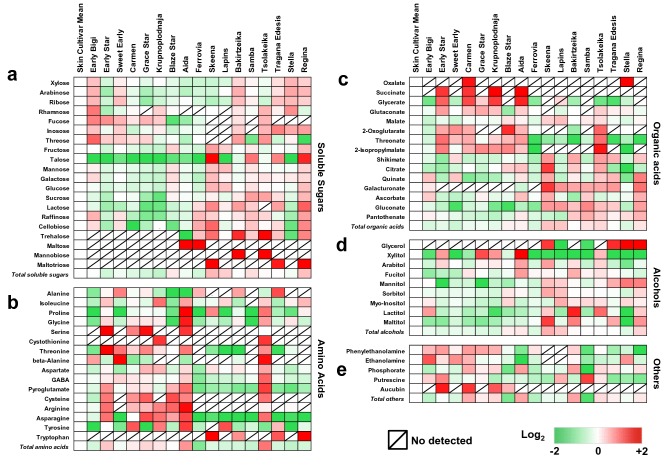
Heat map diagram of primary metabolites in the skin samples of sweet cherry cultivars. Metabolites divided into soluble sugars **a**, amino acids **b**, organic acids **c**, soluble alcohols **d** and other compounds **e**. Based on grand mean log_2_ an increase is depicted as red and a decrease is depicted as green (see color scale). Metabolites abundance were expressed as relative abundance compared to internal standard adonitol. Each metabolite represented by 20 sweet cherry skins in three biological replications. Data are provided in Additional file 3: Table S3

As it is challenging to metabolically monitor the factors involved in fruit cracking, we next studied whether the skin-derived metabolite profiling could be an effective tool to understand this physiological disorder, using GC–MS analysis. Sixty-five polar metabolites in the skin tissue of the seventeen sweet cherry cultivars were quantified prior to the application of both cracking assays (Additional file 3: Table S3). In terms of chemical composition, and considering all cultivars analyzed, skin metabolomic profile includes soluble sugars (twenty), sugar alcohols (nine), organic acids (fifteen), amino acids (sixteen) and other compounds (five) (Fig. 4). Among the skin tissue of the 17 different cultivars screened, the highest contents of soluble sugars, organic acids, amino acids, total alcohols were recorded in ‘Skeena’, ‘Lapins’, ‘Tsolakeika’, ‘Skeena’ cultivars respectively. In cherry skin tissue, glucose and fructose, malate, γ-aminobutyric acid (GABA), and sorbitol represent the main part of sugars, organic acids, amino acids, total alcohols, respectively (Fig. 4, Additional file 3: Table S3). Most metabolites (54% of them) were detected in all cultivars; with some exceptions that were enriched only in specific cultivars. For instance, the highest levels of maltose were detected in ‘Aida’ and ‘Ferrovia’ while mannobiose was exclusively high in two Greek-originated cultivars: ‘Bakirtzeika’ and ‘Tsolakeika’.

The proportions of individual primary metabolites in skin tissue showed significant variation. Six primary metabolites in the skin of ‘Early Bigi’ (oxalate, xylose, arabinose, ribose, mannitol and myo-inositol) as well as in the skin of ‘Samba’ (putrescine, galactose, gluconate, pantothenate, sucrose and lactose) had the highest concentration, respectively. In addition, ethanolamine, rhamnose, fucose, threose and raffinose were most abundant in ‘Stella’. Cultivar ‘Early Star’ was particularly rich in phenylethanolamine, serine, threonine and aucubin (from other compounds) while ‘Bakirtzeika’ displayed high levels of alanine, phosphorate, lactitol and mannobiose. Furthermore, β-alanine, aucubin, maltose, inosose and asparagine had the highest abundance in the skin of cultivars ‘Sweet Early’, ‘Carmen’, ‘Ferrovia’, Tragana Edessis’ and ‘Grace Star’, respectively (Fig. 4, Additional file 3: Table S3). Isoleucine, succinate and aucubin were highly accumulated in ‘Krupnoplodnaja’. The cultivar with the highest malate, sorbitol and tyrosine was the ‘Lapins’ while ‘Regina’ showed the highest levels of glycerol and quinate (Fig. 4, Additional file 3: Table S3). Much higher levels of proline, glycine, glycerate, arginine, xylitol, arabitol, fucitol and ascorbate were found in the skin of ‘Aida’. Cystothionine, glutaconate, aspartate, 2-oxoglutarate, GABA, pyroglutamate, threonate, cysteine, 2-isopropylmalate and trehalose had the highest concentration in ‘Tsolakeika’. Statistically highest contents of eleven primary metabolites, namely fructose, shikimate, citrate, talose, mannose, glucose, galacturonate, tryptophan, cellobiose, maltitol and maltotriose were measured in ‘Skeena’ (Fig. 4, Additional file 3: Table S3).

### Skin secondary metabolites profile among cultivars

**Fig. 5:**
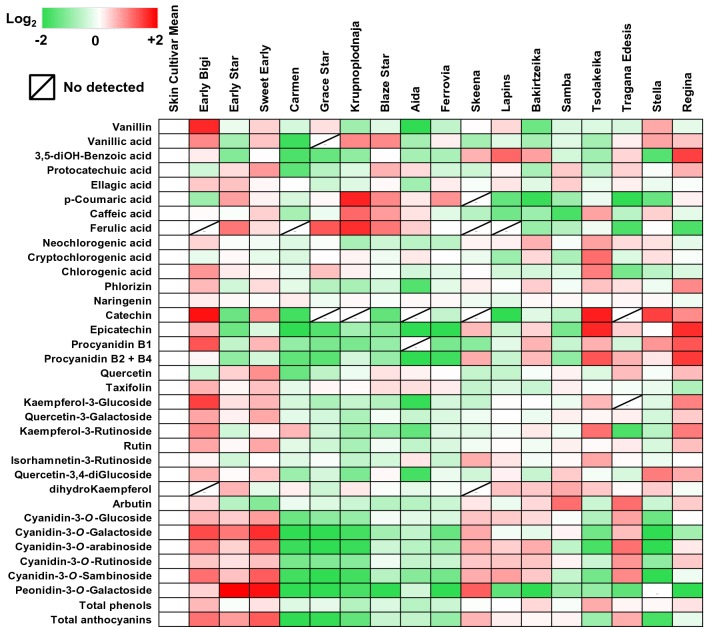
Heat map diagram of polyphenolic compounds in the skin samples of sweet cherry cultivars. Based on grand mean log_2_ an increase is depicted as red and a decrease is depicted as green (see color scale). Metabolites abundance were expressed as mg 100 g^−1^ freeze dried tissue. Each metabolite represented by thirty sweet cherry skins in three biological replications. Data are provided in Additional file 5: Table S4

Another major target of interest in this study was the patterns of metabolites associated with secondary metabolism in the various cherry cultivars. The UPLC–MS/MS analysis confirmed the presence of thirty-five polyphenolic compounds in the skin samples (Additional file 5: Table S4); these secondary metabolites correspond to six anthocyanins and twenty-nine other polyphenols (Fig. 5). Generally, the ‘Krupnoplodnaja’ had the lowest content of total polyphenols contrary to ‘Tsolakeika’, which presented significant enrichment in these compounds (Fig. 5, Additional file 5: Table S4). Also, the content of skin anthocyanins was higher in ‘Sweet Early’ and lower in ‘Carmen’ (Fig. 5, Additional file 5: Table S4).

Significant differences in the polyphenolic composition of the cultivars were noted (Fig. 5). For instance, the polyphenolic compounds vanillin, vanillic acid, naringenin, catechin, procyanidin B1, taxifolin (syn. dihydroquercetin), kaempferol-3-*O*-glucoside, quercetin-3-*O*-galactoside and rutin (syn. quercetin-3-*O*-rutionisde) had the highest concentration in the skin of ‘Early Bigi’. The ‘Sweet Early’ skin contained significantly higher levels of protocatechuic acid, quercetin, cyanidin-3-*O*-glucoside, cyanidin-3-*O*-galactoside, cyanidin-3-*O*-arabinoside and cyanidin-3-*O*-sambinoside. Also, neochlorogenic acid, cryptochlorogenic acid, chlorogenic acid, epicatechin, kaempferol-3-*O*-rutinoside and isorhamnetin-3- *O*-rutinoside were more abundant in skin of ‘Tsolakeika’ (Fig. 5, Additional file 5: Table S4). The cultivar ‘Krupnoplodnaja’ exhibited the highest concentration in *p*-coumaric acid, caffeic acid and ferulic acid. Very high levels of 5-dihydrobenzoic acid, phloridzin and procyanidin B2 + B4 were measured in ‘Regina’. In addition, the cultivars ‘Early Star’ and ‘Samba’ had the highest abundance in ellagic acid and peonidin-3-*O*-galactoside as well as in dihydrokaempferol and arbutin, respectively (Fig. 5, Additional file 5: Table S4). The cultivars ‘Carmen’, ‘Tragana Edessis’ and ‘Stella’ contained considerably higher amounts of naringenin, cyanidin-3-*O*-rutinoside and quercetin-3,4-*O*-diglucoside (Fig. 5, Additional file 5: Table S4).

### Physiological traits and skin metabolites in relations to cracking

**Fig. 6:**
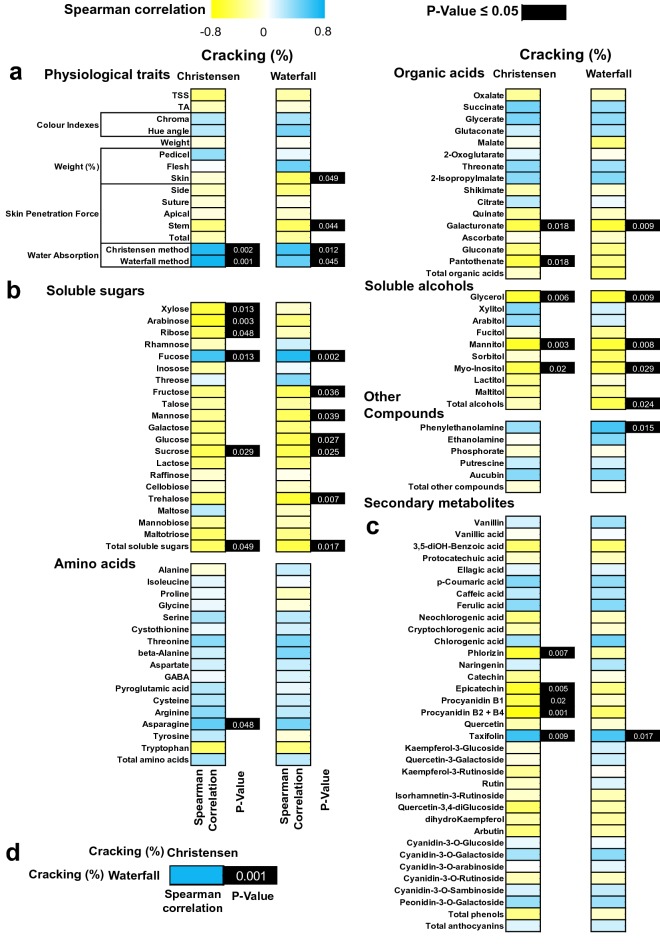
Heat map diagram of spearman correlation between the two cracking assessment methods (Christensen and Waterfall). Physiological traits **a**, primary polar metabolites **b** and secondary metabolites **c**. Correlation between Christensen and Waterfall methods regarding cracking index evaluation **d**. Positive correlation is depicted as blue and negative correlation is depicted as yellow (see color scale). Black color in boxes of *p* value indicate significance of tested correlation

To elucidate the connection between skin metabolites and cracking in different cultivars, we conducted a correlation analysis. Highly positive correlations were observed between water absorption and skin cracking index assessment using both Christensen and Waterfall methods, the statistical significance was cultivar-specific as depicted at Fig. 6a. Furthermore, a strong negative correlation was detected for skin samples between cracking index and two physiological traits skin weight and penetration force around the fruit stem (Fig. 6a). The metabolites sucrose, total soluble sugars (Fig. 6b), galacturonate (Fig. 6b), glycerol, mannitol, *myo*-inositol (Fig. 6b) were negatively correlated with cracking assessed with both tested methods. Interestingly, the compound fucose (Fig. 6b) and taxifolin (Fig. 6c) displayed the strongest positive correlation with the cracking index, as evaluated by both assays. Also, negative correlation was recorded between the metabolite’s xylose, arabinose, ribose (Fig. 6b), pantothenate (Fig. 6b), phloridzin, epicatechin, procyanidin B1 and procyanidin B2 + B4 (Fig. 6c) with cracking index using the Christensen method. On the other hand, positive correlation was detected between asparagine and cracking following Christensen method (Fig. 6b). Using the Waterfall method, negative correlation among fructose, mannose, glucose, trehalose (Fig. 6b), total alcohols (Fig. 6b) and cracking index was determined. In addition, the cracking index based on Waterfall method was positively correlated with phenylethanolamine (Fig. 6b). It was worth noting that the two methods showed a strong positive correlation regarding the cracking index (Fig. 6d).

## Discussion

Fruit cracking is a serious economic problem in sweet cherry production worldwide [10]. This phenomenon is mainly caused by rainfall during the harvest period and it is related to osmotic influences and fruit water permeability [21]. Moreover, factors involved in the differential resistance among cultivars are still unknown [11]. To characterize sweet cherry cracking, we used seventeen cultivars with high commercial value [22] that exhibited different susceptibility to cracking, including relative cracking tolerant (e.g., Regina) and cracking semi-tolerant (e.g., Lapins) cultivars [3]. All cultivars were sampled at commercial harvest stage (Figs. 1 and 2) and afterwards were directly assessed in the laboratory for cracking by submerging their fruit in distilled water (Christensen method) [5]. However, this method has caused concerns in the scientific community because the immersion of sweet cherries to water alters their ionic balance and disturbs the skin surface permeability; as a result in situ cracking conditions may differ from in vitro cracking [23]. To overcome this limitation, we designed the Waterfall method, which allows to simulate the deposition of water on the fruit after the rainfall, thereby create artificial in vitro environment that simulate natural rainfall-induced cracking conditions in a fully automated manner.

Our experimental results showed that the expression of main cracking symptoms undergo variations depending on the evaluation method applied (Fig. 3d). As an example, we noticed that the main type of cracking in the cultivars ‘Early Bigi’ and ‘Early Star’ was near the stem according to Christensen method (Fig. 3d). However, in the same cultivars the cracking was expressed near the fruit apical using the Waterfall method (Fig. 3d). Current data further revealed that Christensen method exhibited lower distinctive ability for cracking evaluation compared to Waterfall method. Indeed, Christensen method grouped eleven cultivars with cracking index higher than 70% (Fig. 3b) while Waterfall method listed five cultivars with cracking index higher than 50% (Fig. 3b). These data illustrated that six cultivars (‘Early Star’, ‘Blaze Star’, ‘Aida’, ‘Ferrovia’, ‘Skeena’, ‘Lapins’) that grouped as relative cracking-susceptible following Christensen method they were also characterized as relative cracking-resistant based on Waterfall method (Fig. 3b).

Sweet cherries water absorption at harvest could be influenced by water temperature, water potential [24], cuticle composition [11] and mineral concentration of water [25]. In addition to this, current data underlying the significance of genotype in water absorption from sweet cherry fruit. In both methods a sharp absorption of water was determined in cultivars ‘Krupnoplodnaja’ and ‘Aida’ (Fig. 3c). Fruit water absorption overcame 4% in cultivars ‘Tsolakeika’ and ‘Grace Star’ estimated by Christensen method (Fig. 3c), while water absorption was determined higher than 2% of total weight in ‘Sweet Early’ assessed by Waterfall method (Fig. 3c).

As skin metabolism is closely linked to cracking [26], our study used central metabolism analysis in skin samples of the different cultivars to unravel these relationships with cracking index. Notably, this analysis revealed that trehalose was not detected in cracking-sensitive cultivars but was highly accumulated in the cracking-resistant ones (Fig. 4a). In addition, trehalose was strongly correlated with cracking as determined by Waterfall method (Fig. 6b), indicating that the cultivars with higher concentration of trehalose are more resistance to cracking. Trehalose production seems to be exclusively reserved for stress resistant plants living under unfavorable situations [27], including water stress conditions. Increasing evidence defines that trehalose metabolism is important for improved stress tolerance. Indeed, overexpression of trehalose biosynthetic genes results in a more sensitive reaction of the stomatal guard cells and closing of the stomata under water stress conditions [28]. Interestingly, an up-regulation of several genes involved in trehalose metabolism has been observed in African Pride atemoya during various cracking stages [29]. Taking into account that the osmotic water uptake through the skin is an important factor for rain-induced sweet cherries cracking [30] along with the excellent capacity of trehalose to protect membranes and proteins from degradation [31], we assume that trehalose could not be only directly involved in cracking but can also regulate this process mediating the crosstalk with osmoprotectant-related compounds as, for example, with sugars [32]. In support to this hypothesis, sucrose as well as several soluble sugars, such as glucose and fructose are negatively correlated with cracking (Figs. 4a and 6b, respectively). The accumulation of these sugars in fruit skin plays an osmoregulatory role and would decrease the fruit permeability, thereby would allow less water entry in sweet cherry fruit when exposed to water stress conditions, such as rain-induced cracking [11].

Another interesting finding that emerged from this work is the fact that the neutral sugars xylose and arabinose displayed a negative correlation with cracking symptoms, as evaluated by Christensen method (Fig. 6b). A clear connection among xylose, arabinose and cracking expression patterns in sweet cherry has been recently established [13], further supporting the role of these metabolites in fruit cracking. Our data also demonstrated that several soluble alcohols such as glycerol, mannitol and inositol, were negatively correlated, independently of the applied method, with cracking rate (Figs. 4d, 6b). It was noteworthy that in both cracking assessment methods galacturonate, which participate in high levels on pectin formation [33], was negatively correlated with cracking (Fig. 6b). The accumulation of galacturonate in skin tissues could be associated with the activation of *β*-galactosidase and the solubilization of pectin [34]. This observation is consistent with results previously obtained [35], who found that the up-regulation of various cell-wall genes, such as *β*-galactosidase in sweet cherry fruit contribute to a greater flexibility and elasticity of the skin, which in turn is reflected by a lower percent of cracking. In this regard, cracking-susceptible sweet cherry cultivars release higher level of soluble pectin fractions during hydrocooling conditions [25]. In line to this, the high level of galacturonate in cracking-resistant cultivars (Fig. 4c) could also be advocated as a precursor compound for protopectin, which may contribute to early pectin formation [29]. Therefore, factors influencing galacturonate metabolism could also influence the extent of fruit cracking. In parallel, fucose that also participates in pectin structure [36] and metabolically is incorporated into cell walls  [37], has shown a strong positive correlation with the cracking assessment using both assays (Fig. 6b), proposing that pectin metabolism is linked to cracking phenomenon. To this end, we noted that pantothenate and asparagine were both correlated with cracking determined by Christensen method (Fig. 6b). Pantothenate (vitamin B5) is the precursor for the synthesis of enzyme co-factors essential for key metabolic and energy-yielding pathways like fatty acids metabolism [38]. Asparagine is a reservoir for nitrogen in plants and is being accumulated under adverse conditions such as biotic and abiotic stresses [39]. However, further research is needed to unravel the function of these metabolites in cracking.

In addition to primary metabolism, we also targeted pathways associated with secondary metabolites, such as polyphenolic compounds, that are known to be important in sweet cherry fruit biology [12, 15]. Earlier study [40] revealed a high variability of secondary metabolites among different sweet cherry cultivars. In the current study, thirty-five phenolic compounds, including six anthocyanins and others phenolic classes were determined in cherry skin samples at various concentration (Fig. 5, Additional file 5: Table S4). Previous work pinpointed the high connectivity of polyphenolic compounds with cracking-sensitive citrus cultivars [41]. Accordingly, our results disclosed that several polyphenols, such as phloridzin, epicatechin, procyanidin B1 and B2 + B4 showed a negative correlation with the cracking using Christensen method (Fig. 6c), suggesting that this physiological disorder is controlled to a high degree of the cultivar-specific polyphenolic metabolic regulation.

In this study one curious observation is the fact that the cracking index was positively correlated with the flavonoid taxifolin in the skin of sweet cherry cultivars, irrespectively of the tested method (Fig. 6), might signifying that taxifolin could be considered as a marker of fruit cracking. Taxifolin (3,5,7,3,4-pentahydroxyflavanone or dihydroquercetin) accumulates to high levels in grape and oranges fruits [42] and many studies have pointed out that taxifolin possesses strong anti-oxidant and anti-radical activity in various cell systems [43, 44]. However, as taxifolin is a common intermediate in the flavonoid/anthocyanin pathway and is only detected in minor concentration (compared to that found in other plant species cited here) is unlikely to have this effect on cracking and/or need more detailed studies to elucidate its function. Given that the excessive water uptake in fruit during the cracking process induced the generation Reactive Oxygen Species (ROS) [45], this study could suggest that taxifolin but also other polyphenolic compounds found here has the potential to diminish cracking levels in cherry skin, possibly by scavenging ROS.

## Conclusion

The current data indicated that the proposed Waterfall assay could be more reliable than the Christensen method for determining the skin cracking of sweet cherry cultivars. This is supported by the fact that this assay displayed high negative correlation between skin weight and cracking index (Fig. 6a), a situation that can mimic the rain-induced fruit cracking under field conditions. In addition, a negative pattern between cracking assessment and skin penetration force near stem were observed using the Waterfall approach (Fig. 6a). Hence, Waterfall method could provide a more effective cracking-based classification of cherry cultivars. Moreover, the strong positive correlation between both assays (Fig. 6d), indicate the existence of a significant relationship between the two methods, as also demonstrated by the relative common pattern of cracking classification among several cultivars (Fig. 6). Despite all these, the most important advantages of Christensen method remain that this assay is short-term, time-saving and suitable for automation. Based on insights gained in this study, we conclude that a combination of both assays (Christensen and Waterfall methods) enables considerably more robust and accurate cracking results to be obtained when compared to single-test using one method, at least for the studied cultivars. This combined laboratory-based methodology allows a fast screen of many different cultivars, in order to select potential candidates to be cracking-resistant, avoiding long and expensive field tests. We also investigated whether the metabolic composition of skin tissue among the selected cultivars would have an impact on cracking index tested by the two assays. This metabolic approach suggests that changes in the content of a few metabolites, such as fucose and maybe taxifolin (or other polyphenols), could be correlated with the observed difference in cracking of the cultivars studied. Future investigations may focus on dissecting the metabolome of sweet cherry cultivars at different developmental stages, as well as on the connection of the metabolic variations to genomic/proteome changes. Altogether, this work sheds insight on the cracking evaluation process in cherry fruit and provides novel information that may be used in potential molecular breeding efforts to improve sweet cherry fruit resistance to cracking in the future.

## Supplementary information

**Additional file 1: Table S1.** Physiological traits of seventeen cherry cultivars at harvest and MANOVA output.

**Additional file 2: Table S2.** Texture properties, main cracking of cultivars at harvest and MANOVA output.

**Additional file 3: Table S3.** Quantitative results of skin primary metabolite analysis and MANOVA output.

**Additional file 4: Fig. S1.** Large-scale spearman correlation analysis.

**Additional file 5: Table S4.** Quantitative results of skin secondary metabolite analysis and MANOVA output.

## Data Availability

All data generated in this study are included in this article and additional files. Material is available from the corresponding author on reasonable request.
