# Supplementary figures and images for: Sweet cherry fruit cracking: follow-up testing methods and cultivar-metabolic screening
Source: Plant Methods. 2020 Apr 10;16:51. doi: 10.1186/s13007-020-00593-6 (PMC7149889; doi:10.1186/s13007-020-00593-6)

## Slide 1
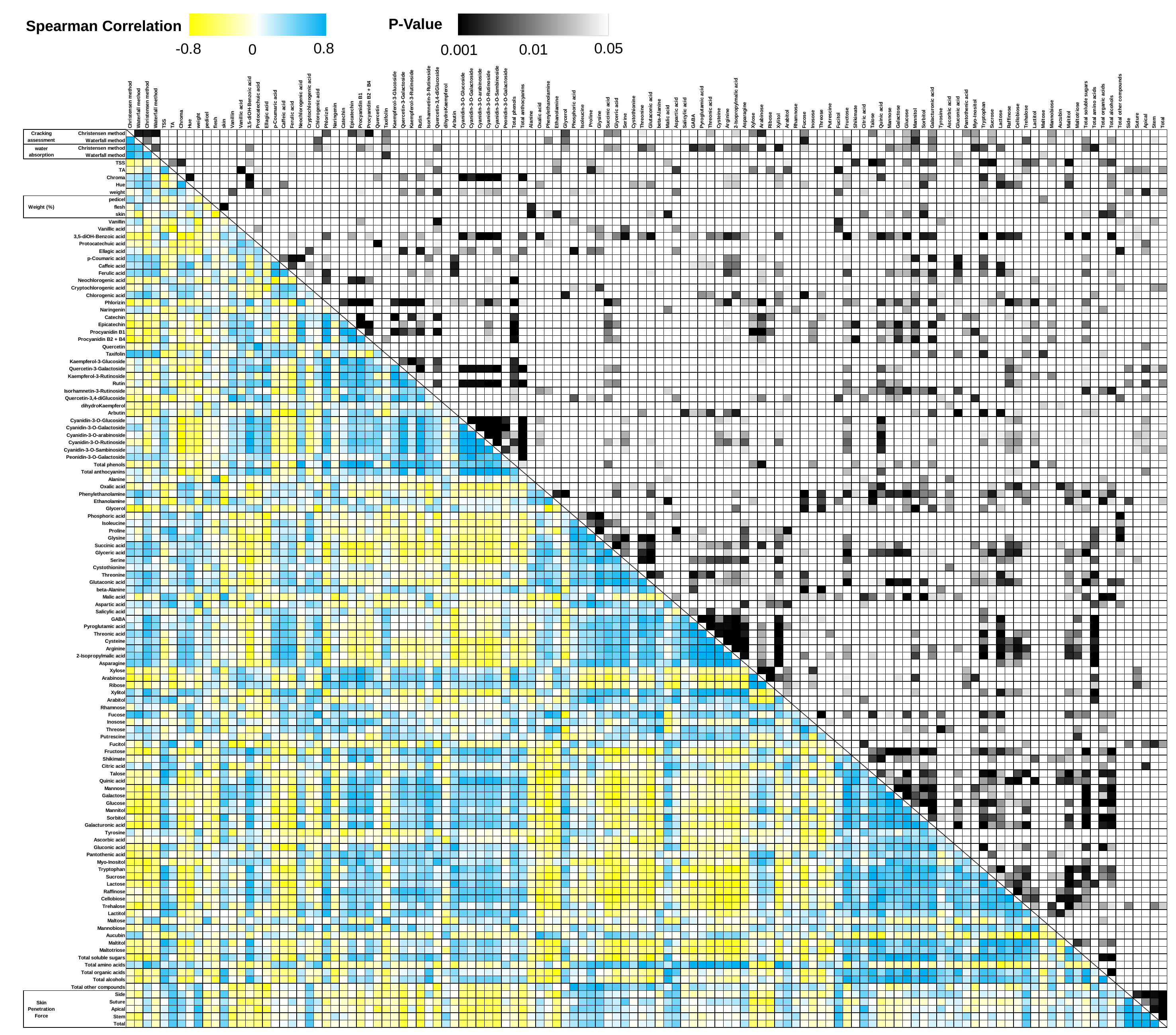

P-Value
Spearman Correlation
0.8
0.05
-0.8
0
0.01
0.001

Supplement: Supplementary file 4 — Additional file 4: Fig. S1. Large-scale spearman correlation analysis. [file 13007_2020_593_MOESM4_ESM.pptx]
